# The effectiveness and safety of total glucosides of paeony in systemic lupus erythematosus: A systematic review and meta-analysis

**DOI:** 10.1097/MD.0000000000032029

**Published:** 2022-12-16

**Authors:** Mengjie Wang, Zhiyuan Wang, Ying Liu, Lei Wang, Xiaomeng Wang, Ping Jiang

**Affiliations:** a First Clinical Medical College, Shandong University of Traditional Chinese Medicine, Jinan City, China; b Emergency Department, People’s Hospital of Zhengzhou, Zhengzhou City, China; c Rheumatology and Immunology Department, The Affiliated Hospital of Shandong University of Traditional Chinese Medicine, Jinan City, China.

**Keywords:** Meta analysis, randomized controlled trial, systemic lupus erythematosus, total glucosides of paeony

## Abstract

To evaluate the efficacy and safety of total glucosides of paeony in the treatment of systemic lupus erythematosus (SLE).

**Methods:**

From the creation of the database to July 2021, multiple databases were searched for randomized controlled trials of treating SLE with total glucosides of paeony (TGP) combining chemical medicine. After screening, quality evaluation and data extraction, the included studies were analyzed by Revman5.3 software.

**Results:**

A total of 11 studies were included, including 836 patients (treatment group 417, control group 419). Meta analysis showed that on the basis of routine treatment, TGP could further improve the treatment effective rate (OR* = *4.19, 95% CI: 2.21 to 7.95, *Z *= 4.38, *P < *.0001), reduced SLE Disease Activity Index (SLEDAI) (MD* *= −1.70, 95%CI: −2.51 to −0.89, *Z *= 4.11, *P *< .0001) and erythrocyte sedimentation rate (MD* *= −7.04, 95%CI: −8.48 to −5.59, *Z *= 9.53, *P < *.00001), reduced the level of immunoglobulin A (IgA) (MD* *= −0.60, 95%CI: −0.82 to −0.37, *Z *= 5.24, *P *< .00001), immunoglobulin G (IgG) (MD* *= −2.97, 95%CI: −3.72 to −2.23, *Z *= 7.82, *P < *.00001), and immunoglobulin M (IgM) (MD* *= −0.36, 95%CI: −0.45 to −0.27, *Z *= 7.54, *P *< .00001), increased the level of complement C3 (MD* *= 0.34, 95%CI: 0.30 to 0.39, *Z *= 14.40, *P* < .00001) and complement C4 (*MD *= 0.07, 95%CI: 0.06 to 0.08, *Z *= 10.08, *P* < .00001), and decreased the recurrence (OR* *= 0.31, 95%CI: 0.16 to 0.61, *Z *= 3.39, *P *= .0007), and there was no significant difference in the incidence of adverse reactions (OR* *= 0.93, 95%CI: 0.45 to 1.91, *Z *= 0.20, *P = *.84).

**Conclusion:**

On the basis of conventional treatment, the combined use of TGP can enhance the clinical efficacy of SLE without increasing the incidence of adverse effects.

## 1. Introduction

Systemic lupus erythematosus (SLE) is an autoimmune and systemic disease characterized by complex and varied clinical symptoms. Although fever, rash, butterfly erythema, mucosal ulcers, and arthritis are the characteristic first signs of SLE, the disease can manifest in many unexpected ways. More seriously, abrupt onset with target organ involvement is also common, such as hematologic findings, renal findings, respiratory symptoms, or central nervous system signs. A large number of autoantibodies in SLE is characterized by exaggerated B cell and T cell responses and loss of immune tolerance against self-antigens.^[1]^ The disease tends to occur in women of childbearing age, and women are more prone to develop SLE than men, with a sex ratio of 6:1.^[2,3]^ The prognosis of men is often worse than that of women.^[4]^

At present, there is no cure for SLE, but it can be effectively controlled by delaying the progression of the disease, reducing organ damage, and improving patients’ quality of life. Medications are mainly concentrated on glucocorticoids, hydroxychloroquine, immunosuppressants, and biologics. Although good clinical efficacy has been achieved^[1]^ but long-term use of glucocorticoids and immunosuppressants may produce serious side effects, and biologics may increase the financial burden.^[5,6]^ In recent years, because of its low side effects and multitarget characteristics, traditional Chinese medicine (TCM) has a good therapeutic prospect and has achieved good efficacy in the treatment of SLE,^[7]^ highlighting its certain advantages.

As the dry root of herbaceous peony in Ranunculaceae, Baishao (Paeonia Lactiflora) has the functions of nourishing blood and consolidating Yin, softening liver and alleviating pain, and inhibiting liver Yang. Studies have demonstrated its Chinese herbal formula and extract have certain effects on the treatment of SLE.^[8,9]^

Total glucosides of paeony (TGP) is the extract of Chinese herbal medicine Baishao. The active ingredient monomers are polyglycosides such as paeoniflorin, hydroxy paeoniflorin, and benzoyl paeoniflorin. The main active ingredient is paeoniflorin, accounting for more than 90% of the total content.^[10]^ TGP has anti-inflammatory, analgesic, hepatoprotective, and autoimmune effects.^[11,12]^ It is a kind of immunomodulator which is widely used in clinic at present and has good curative effect on SLE.^[13]^ However, the safety and effectiveness of TGP in the treatment of SLE have not been systematically evaluated in the past 7 years. Only one paper evaluated its curative effect 7 years ago,^[14]^ but only 2 articles were included in this paper as randomized controlled trials (RCTs) and the total sample size was small, so, the reliability of the results was low. Therefore, in order to develop higher-quality medical evidence based on data, the efficacy and safety of TGP in treating SLE were studied systematically for clinical reference..

## 2. Methods

### 2.1. Search strategy

We searched PubMed (Medline), Embase, Cochrane Central Register of Controlled Trials (CENTRAL), Web of Science, China National Knowledge Infrastructure (CNKI), Wanfang Med Database, Chinese VIP Information Database (VIP), and Chinese Biomedical (CBM) Database from their inception to July 31, 2021. Ongoing surveillance was conducted after July 2021 through December, 2021, to identify newly published studies that might affect the results. The last surveillance on December 01, 2021, identified no new studies. The languages were restricted in English and Chinese. For the English electronic databases, the search strategy was set as follows: (((“Lupus Erythematosus, Systemic”[Mesh]) OR (((((Systemic Lupus Erythematosus[Title/Abstract]) OR (Lupus Erythematosus Disseminatus [Title/Abstract])) OR (Libman-Sacks Disease[Title/Abstract])) OR (Disease, Libman-Sacks[Title/Abstract])) OR (Libman Sacks Disease[Title/Abstract]))) AND ((((total glycosides of paeony[Title/Abstract]) OR (Paeoniflorin[Title/Abstract])) OR (paeoniflorin sulfonate[Title/Abstract])))) AND (randomized controlled trial [Publication Type] OR randomized[Title/Abstract] OR placebo[Title/Abstract]).

### 2.2. Procedures

The systematic review was registered in PROSPERO (International Prospective Register of Systematic Reviews) (CRD42021270585) and performed in accordance with the Preferred Reporting Item for Systematic Reviews and Meta-Analyses (PRISMA) Statement.

### 2.3. Eligibility criteria

(1) Type of study: Only RCTs on TGP treatment for SLE were eligible.
(2) Type of participants: Adult patients who meet the diagnostic criteria of SLE (e.g., the SLE diagnostic criteria revised by American College of Rheumatology (ACR),^[15,16]^ the diagnostic criteria formulated by Chinese Rheumatology Association,^[17]^ or revised by International Cooperative Clinical Center for SLE,^[18]^ etc.).
(3) Type of intervention: The control group was treated with glucocorticoids and/or immunosuppressants, and TGP was given on the basis of the control group in the experimental group.
(4) Type of results: The main outcome indicators included clinical effective rate^[19]^ and SLE disease activity index (SLEDAI). Secondary outcome indicators: Inflammatory indicators erythrocyte sedimentation rate (ESR), serum immunological indexes, such as complement C3 and C4 (C3, C4), immunoglobulin (IgG, IgA, IgM), recurrence rate, and incidence of adverse reactions.

### 2.4. Exclusion criteria.

(1)There is no clear and recognized diagnostic criteria.
(2) The baseline between groups is inconsistent.
(3) Full text and original text data cannot be obtained.
(4) The outcome indicators are inconsistent.
(5) Repeated published literature.

### 2.5. Characteristics of the included studies and quality assessment

Two researchers independently evaluated the studies based on the inclusion criteria and cross-checked the extracted data. When disagreements arose, 2 authors engaged in discussion to resolve them. If disagreements continued, the third author was consulted for final decisions. Then, 2 researchers independently extracted data from the eligible trials using a template that included personal information, intervention measures, outcome indicators, treatment courses, adverse reactions of patients in experimental and control groups, etc.

The Jadad scale and version 2.0 (RoB2.0) of the risk bias assessment tool released by the Cochrane Methodology Working Group were used for bias risk assessment.^[20]^ RoB2.0 establishes 5 modules to evaluate the risk of bias from various perspectives, namely bias generated by the random process, bias deviating from the established intervention, bias of missing outcome data, bias of outcome measurement, and bias of selective reporting of results. The signal questions in multiple modules were evaluated in accordance with RoB2.0 guidelines. The answer to the signal problem is: Yes (Y), probably yes (PY), probably no (PN), no (N), and no information (NI). The bias risk is subdivided into low risk, moderate risk, and high risk based on signal responses to various modules, and the overall bias risk is also provided.

### 2.6. Statistical analysis

The software Revman 5.3 was used for data analysis. The odds ratio (OR) was used for binary variable data, while mean difference (MD) was used to represent continuous variable data, and the 95% confidence interval (CI) was applied. The heterogeneity was tested by the *I*-squared (*I*^2^) and Chi-squared (*P*) tests. If Chi-squared tests *P *> .1 and *I*^2^ < 50%, the heterogeneity was low and the fixed-effects model is used for analysis; otherwise, the heterogeneity is high, and the random-effects model is used for analysis. We would then conduct sensitivity analysis and subgroup analysis, if necessary, to determine the source of heterogeneity. If the effect value is 10 and is mentioned in the prior literature, a funnel plot should be created to assess the risk of publication bias.

### 2.7. Ethical review

This is a meta-analysis that follows PRISMA principles and therefore does not require ethical review.

## 3. Results

### 3.1. Document retrieval process

**Figure 1.**
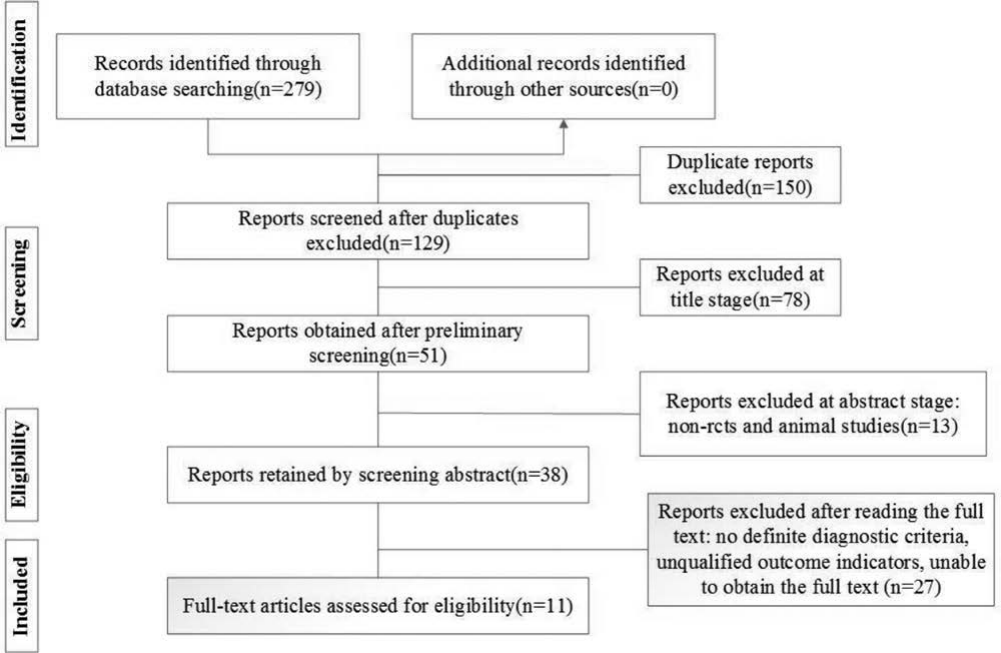
Literature search flow chart.

The bibliographic search identified 279 articles. Eleven studies finally met the inclusion criteria after gradual screening.^[21–31]^ The steps of specific screening trials are presented in Fig. 1.

### 3.2. Characteristics of included literatures

We included a total of 11 literatures,^[21–31]^ including 836 patients, of which 417 is in the treatment group and 419 in the control group. The literatures we included were all domestic studies, not foreign studies. In the literature, the patients in the control group were treated with glucocorticoid and/or immunosuppressant, and TGP were given on the basis of the control group in the experimental group. The baselines of the experimental group and the control group were comparable. The diagnostic criteria of all published works were lucid and trustworthy, and the evaluation criteria of each outcome index were consistent. The main outcome indicators were clinical effective rate and SLEDAI score; Secondary outcome indicators: ESR, IgA, IgG, IgM, C3, C4, recurrence rate, incidence of adverse reactions. The characteristics of studies are shown in Table 1.

**Table 1:** Characteristics of studies.

Included literature	Intervention measures		TGP dosage	Outcome indicators	Course of treatment/month	Adverse reaction		Sample size		Gender (male/female)		Average age/year		Course of disease/Year		RCT (yes/no)	Baseline comparable (yes/no)	Jadad score
	E	C				E	C	E	C	E	C	E	C	E	C			
Zhao et al 2020	TGP + GCS + HCQ	GCS + HCQ	0.6g,tid	②,⑦,⑧,⑩	3	3.77% (2/53)	5.66% (3/53)	53	53	3/50	5/48	38.14 ± 3.24	34.13 ± 3.22	0.83 ± 0.19	0.82 ± 0.19	Yes	Yes	High
Wu et al 2020	TGP + CTX + GCS	CTX + GCS	0.6g,bid-tid	④,⑤,⑥,⑦,⑧,⑨,⑩	3	16.22%(6/37)	13.51%(5/37)	37	37	Oct-27	Sep-28	30.11 ± 6.05	29.12 ± 5.64	3.24 ± 1.20	3.19 ± 1.02	Yes	Yes	High
Xu 2020	TGP + GCS + CTX	GCS + CTX	0.6g,bid-tid	②,⑨,⑩	12	8.33%(3/36)	30.56%(11/36)	36	36	Oct-26	Nov-25	36.82 ± 6.29	36.75 ± 6.23	5.39 ± 1.67	5.25 ± 1.72	Yes	Yes	High
Xue and lv 2019	TGP + HCQ + GCS	HCQ + GCS	0.6g,tid	②,⑦,⑧,⑩	6	3.33%(1/30)	6.67%(2/30)	30	330	Mar-27	Mar-27	38.15 ± 3.20	38.12 ± 3.25	1.81 ± 0.32	1.82 ± 0.35	Yes	Yes	High
Yang and li 2019	TGP + GCS	GCS	0.6g,tid	②,⑩	3	16.98%(9/53)	15.09%(8/53)	53	53	May-48	Mar-50	41.5 ± 4.1	42.7 ± 5.2	3.4 ± 1.1	3.7 ± 1.3	Yes	Yes	High
Li et al 2018	TGP + Tacrolimus + GCS	Tacrolimus + GCS	0.6g,bid	①,②,④,⑤,⑥,⑦,⑧,⑨,⑩	6	15.56%(7/45)	11.11%(5/45)	45	45	Sep-36	Jul-38	32.15 ± 5.37	33.21 ± 4.94	2.77 ± 0.41	2.67 ± 0.82	Yes	Yes	High
Cai 2017	TGP + GCS + CTX	GCS + CTX	0.6g,tid	②,③,⑦,⑩	6	6.67%(2/30)	23.33%(7/30)	30	30	May-25	Apr-26	33.3 ± 5.1	34.7 ± 4.5	2.47 ± 0.78	2.56 ± 0.82	Yes	Yes	High
Shao 2017	TGP + GCS	GCS	0.6g,tid	①	3	-	--	25	25	Nov-14	Oct-15	66.5 ± 10.3	63.5 ± 12.3	-	-	Yes	Yes	High
Mo and chen 2014	TGP + GCS	GCS	0.6g,tid	①,②,③,⑦,⑩	3	16.13%(5/31)	12.90%(4/31)	31	31	Oct-52		31.28 ± 12.5		4.56 ± 2.1		Yes	Yes	Low
Sun 2013	TGP + GCS + CTX	GCS + CTX	0.6g,tid	①,②,⑥,⑦	3	-	--	48	48	-	-	-	-	-	-	Yes	Yes	Low
Shuai et al 2003	TGP + GCS	Amylum + GCS	0.6mg,tid	②,③,⑩	3	41.38%(12/29)	3.23%(1/31)	29	31	0/29	Feb-29	31.94 ± 10.80	32.74 ± 7.62	1.95 ± 1.51	2.40 ± 2.02	Yes	Yes	High

Outcome indicators: ①Quality of included studies; ②SLEDAI assessment; ③ESR; ④IgA; ⑤IgM; ⑥IgG; ⑦C3; ⑧C4; ⑨recurrence rate; ⑩ incidence of adverse reactions.

C = control group, CTX = cyclophosphamide, E = experimental group, ESR = erythrocyte sedimentation rate, GCS = glucocorticoid, HCQ = hydroxychloroquine, IgA = immunoglobulin A, IgM = immunoglobulin M, IgG = immunoglobulin G, TGP = total glucosides of paeony.

### 3.3. Quality of included studies

A total of eleven studies were included,^[21–31]^ all of which were RCTs. Nine studies^[21–28,31]^ used the random number table method, and 2 studies^[30,31]^ only referred to randomization, but did not describe the random method.

The researchers of 10 literatures^[21–28,30,31]^ did not report whether there was deviation due to the experimental environment. One^[31]^ was a random double-blind control experiment. So, it was at low risk of bias.

All the 10 articles^[21–30]^ were able to obtain all the data of the subjects, which was at low risk of bias. One literature^[31]^ cannot obtain the data of all subjects. So, it was at high risk of bias. The proportion of data lost between different intervention groups was different, which suggested that the lack of results data had a risk of deviation, so it was at high risk of bias.

Ten articles^[21–28,30]^ did not mention blinding, but the author’s knowledge of intervention status could have influenced outcome assessment but there was no reason to believe that it did. So, it was at moderate risk of bias. One study^[31]^ was a randomized double-blind controlled experiment and was therefore at low risk of bias.

The included literatures did not mention other sources of bias.^[21–31]^ So, they were evaluated as moderate risk of bias.

**Figure 2.**
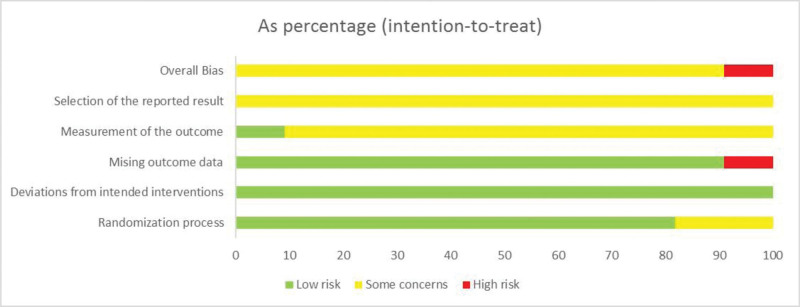
Risk of bias graph of included studies.

A total of 10 studies were at moderate risk of bias,^[21–30]^ and one was at high risk of bias^[31]^(Fig. 2).

### 3.4. Clinical effective rate

**Figure 3.**
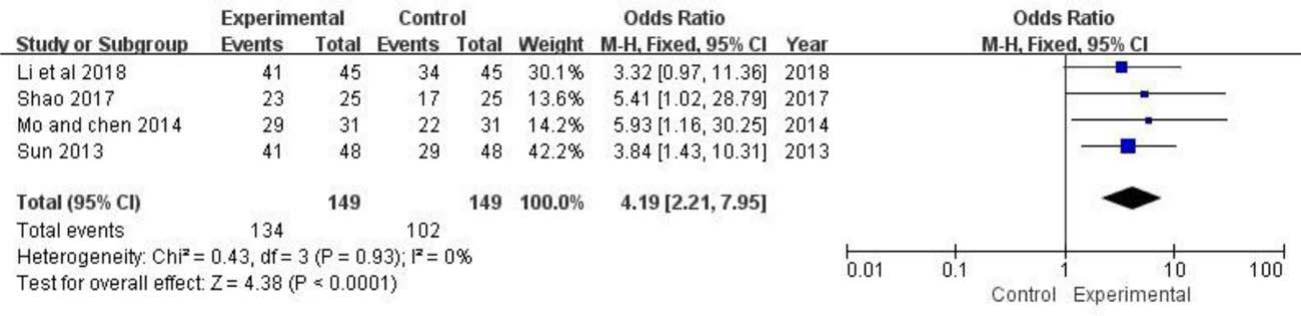
The effect of TGP on clinical effective rate. TGP = total glucosides of paeony.

Four trials reported the clinical effective rate (n = 298).^[26,28–30]^ The fixed-effect model analysis was selected according to heterogeneity test (*P *= .93, *I*^2* *^*= *0%), and results across 4 trials were consistently in the direction of a good curative benefit of daily TGP use. It is suggested that the combination of conventional western medicine and TGP can further improve the clinical efficiency (OR* *= 4.19, 95% CI: 2.21 to 7.95, *Z *= 4.38, *P *< .0001) (Fig. 3).

### 3.5. SLEDAI assessment

SLEDAI scores of 8 trials were analyzed,^[22,23,25–27,29–31]^ including 652 patients.

**Figure 4.**
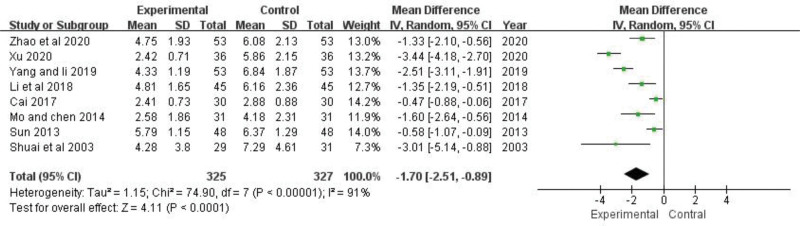
The effect of TGP on SLEDAI score. TGP = total glucosides of paeony.

There was significant heterogeneity between trials (*P *< .00001, *I*^2* *^= 91%). After sensitivity analysis, heterogeneity still persisted within each subgroup, so other sources of heterogeneity were also investigated. Intervention measures may contribute to heterogeneity in SLEDAI scores, which were higher in control group patients than in patients with TGP (MD = −1.70, 95% CI: −2.51 to −0.89, *Z *= 4.11, *P *< .0001) (Fig. 4). The combination of TGP and conventional western medicine treatment tended to yield more conservative results on reducing the SLEDAI score than those obtained by applying conventional western medicine.

In order to find the causes of heterogeneity, they were divided into TGP + GCS, TGP + GCS +  cyclophosphamide (CTX), and TGP + GCS + other for subgroup analysis according to the different intervention measures. The heterogeneity test (*P *< .00001, *I*^*2 *^= 91%) showed that there was great heterogeneity among subgroups, indicating that TGP combined with different drugs for SLE had an effect on heterogeneity.

Among them, there were 3 trials^[25,29,31]^ comparing TGP + GCS with TGP alone, involving 228 patients. Moreover, the SLEDAI score was significantly different between the pooled results. *MD *= −2.28, 95% CI: −2.94 to −1.62, *Z *= 6.67, *P *< .00001). The heterogeneity of the SLEDAI score is still high (*P *= .27, *I*^*2 *^= 24%). The results showed that the SLEDAI score of TGP group was lower than that of the control group.

Three trials^[22,27,30]^ compared TGP + GCS + CTX with GCS + CTX. Based on the analysis, large heterogeneity (*P *< .00001, *I*^*2 *^= 96%) could be attributed to different courses of treatment. The random-effect model was used. The pooled results showed that the SLEDAI score of TGP group was lower than that of the control group, but there was no significant difference (MD* *= −1.47, 95% CI: −3.00 to -0.07, *Z *= 1.87, *P *= .06).

**Figure 5.**
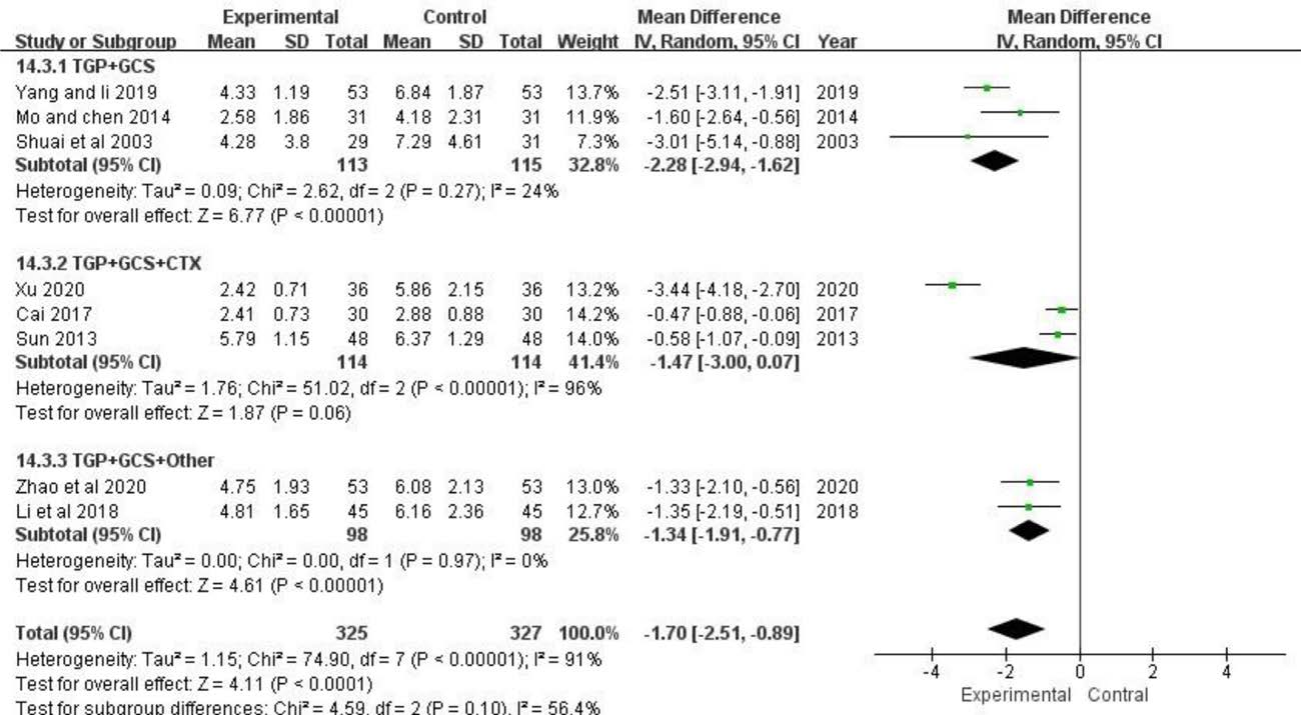
Subgroup analysis of the effect of TGP on SLEDAI score. SLEDAI = SLE Disease Activity Index; TGP = total glucosides of paeony.

Two trials^[23,26]^ reported GP + GCS + other group, involving 196 patients. The heterogeneity test showed that there was no heterogeneity (*P *= .97, *I*^*2 *^= 0%). The comparison of SLEDAI score of TGP + GCS + other group with GCS + other group showed significant differences (*MD *= −1.34, 95% CI: −1.91 to −0.77, *Z *= 4.61, *P *< .00001) (Fig. 5). It is hypothesized that TGP combined with different chemical medicines can reduce the SLEDAI score, but there may be variations; if the sample size is too small, there is a greater chance of bias.

### 3.6. ESR

**Figure 6.**
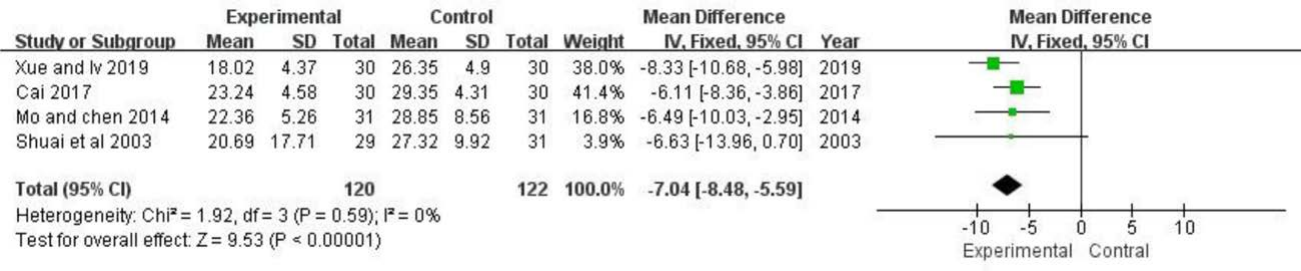
The effect of TGP on ESR. ESR = erythrocyte sedimentation rate, TGP = total glucosides of paeony.

Four of the included trials reporting ESR were conducted (n = 242).^[24,27,29,31]^ The fixed-effect model was adopted for ESR analysis because of its heterogeneity (*P *= .59, *I*^*2 *^= 0%). The pooled analysis showed that ESR of experimental group was significantly reduced compared with control group (*MD *= −7.04, 95% CI: −8.48 to −5.59, *Z *= 9.53, *P *< .00001) (Fig. 6). It is suggested that TGP can further reduce ESR in patients with SLE.

### 3.7. IgA, IgM, and IgG

**Figure 7.**
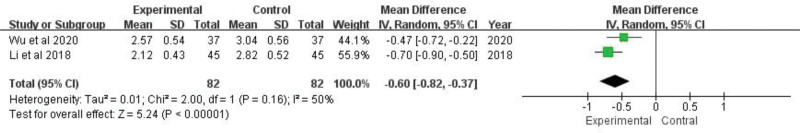
The effect of TGP on IgA. IgA = immunoglobulin A; TGP = total glucosides of paeony.

**Figure 8.**
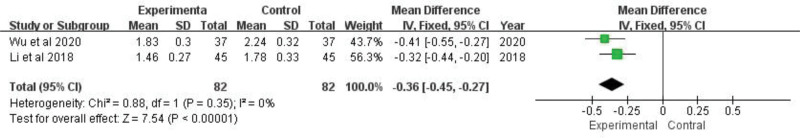
The effect of TGP on IgM. IgM = immunoglobulin M; TGP = total glucosides of paeony.

As per the combined analysis of 2 included trials,^[21,26]^ According to the heterogeneity test (*P* = .16, *I*^2^ = 50%), the IgA data were analyzed using the random-effect model, whereas the IgM data lacked heterogeneity (*P* = .35, I2 = 0%), so the fixed-effect model was utilized. There was significant difference in IgA and IgM of the experimental group and control group (IgA: MD = −0.60, 95% CI: −0.82 to −0.37, *Z *= 5.24, *P *< .00001; IgM: MD* *= −0.36, 95% CI: −0.45 to −0.27, *Z *= 7.54, *P *< .00001) (Figs. 7 and 8), suggesting TGP group has better effect in reducing the level of IgA and IgM.

**Figure 9.**
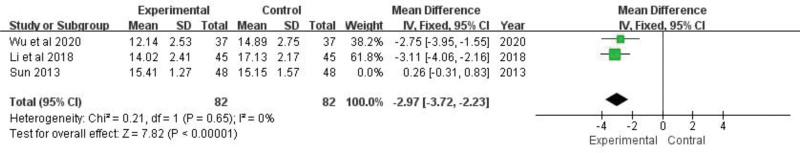
The effect of TGP on IgG. IgG = immunoglobulin G; TGP = total glucosides of paeony.

Three studies^[21,26,31]^ reported the level of IgG, and there was heterogeneity between studies (*P *< .00001, *I*^2* *^= 96%). After sensitivity analysis, it was determined that one of the articles had a greater impact on heterogeneity; however, there was no statistically significant difference between the study and other studies in terms of baseline intervention measures, dose, and course of treatment. The reasons of heterogeneity remained unclear, and then heterogeneity within studies disappeared after the removal of 1 outlier study (*P *= .65, *I*^2* *^= 0%). Therefore, the fixed-effect model was used for analysis. The results showed that TGP decreased the level of IgG better than the control group, with statistical difference (*MD *= −2.97, 95% CI: −3.72 to −2.23, *Z *= 7.82, *P *< .00001) (Fig. 9). It is suggested that the use of TGP can further reduce the IgG level of patients with SLE.

### 3.8. C3, C4

**Figure 10.**
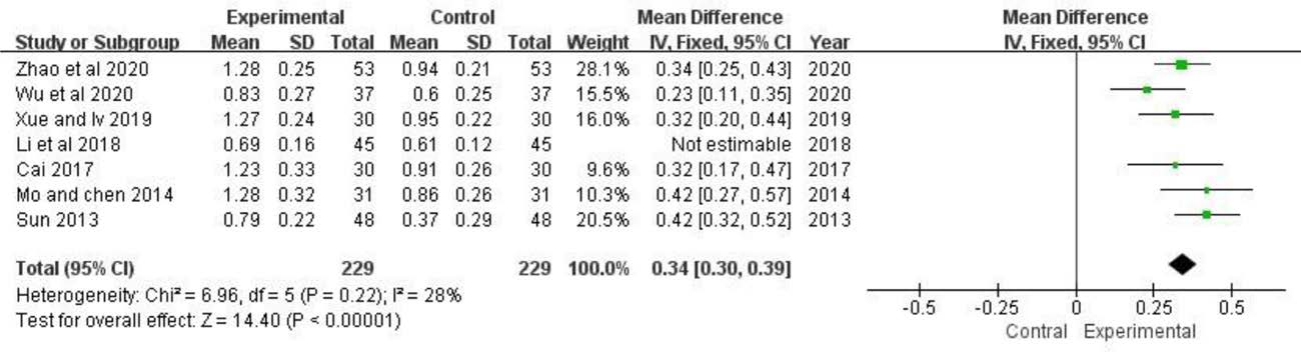
The effect of TGP on C3. TGP = total glucosides of paeony.

**Figure 11.**
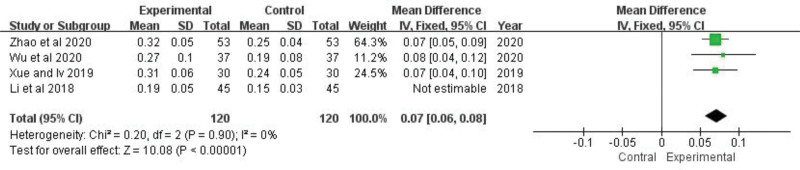
The effect of TGP on C4. TGP = total glucosides of paeony.

C3 of 7 trials^[21,23,24,26,27,29,30]^ and C4 of 4 trials^[21,23,24,26]^ were analyzed, respectively. The heterogeneity test indicated that there was heterogeneity between 2 of them (C3:*P *< .00001, *I*^*2 = *^89%; C4: *P *= .05, *I*^*2 *^= 62%). After sensitivity analysis, it was found that the one of the trials have a greater impact on heterogeneity. The levels of C3 and C4 are easily affected by blood collection time, detection methods, measuring instruments, reagents, and kits. We think that this may be part of the reason for the differences. After deleting the study, there is no heterogeneity (C3: *P *= .22, *I*^2 ^= 28%; C4: *P *= .90, *I*^2* *^= 0%), and then a fixed-effect model analysis was utilized. The results showed that the effects of the experimental group in increasing C3 and C4 were better than that of the control group, which was statistically different (C3: *MD *= 0.34, 95% *CI*: 0.30 to 0.39, *Z *= 14.40, *P *< .00001; C4: *MD *= 0.07, 95% *CI*: 0.06 to 0.08, *Z *= 10.08, *P *< .00001) (Figs. 10 and 11). The pooled analysis showed that C3 and C4 of TGP group was significantly increased compared with control group.

### 3.9. Recurrence rate

**Figure 12.**
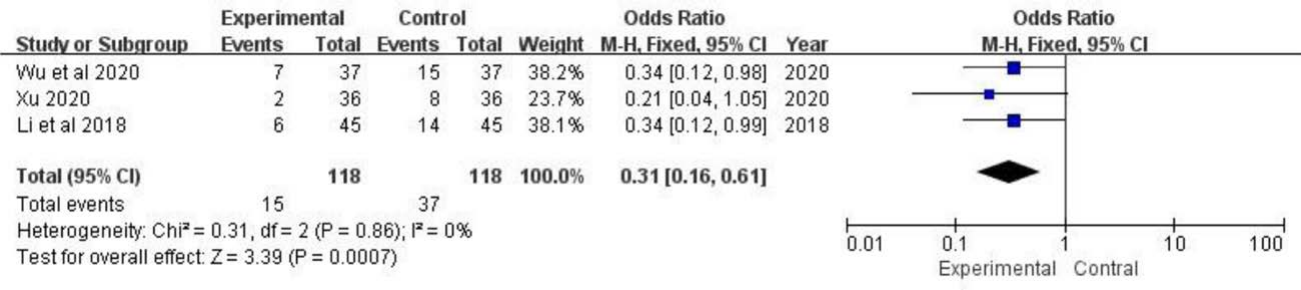
The effect of TGP on recurrence rate. TGP = total glucosides of paeony.

A total of 52 of 236 patients from only 3 studies^[21,22,27]^ described the recurrence rate. The fixed-effect model was applied based on the heterogeneity test (*P *= .86, *I*^2* *^= 0%). The results showed that the experimental group were better than the control group in reducing the recurrence rate, which was statistically different (OR = 0.31, 95%CI: 0.16 to 0.61, *Z *= 3.39, *P *= .007) (Fig. 12). It was discovered that the inclusion of TGP considerably lowered the recurrence rate.

### 3.10. Safety evaluation

**Figure 13.**
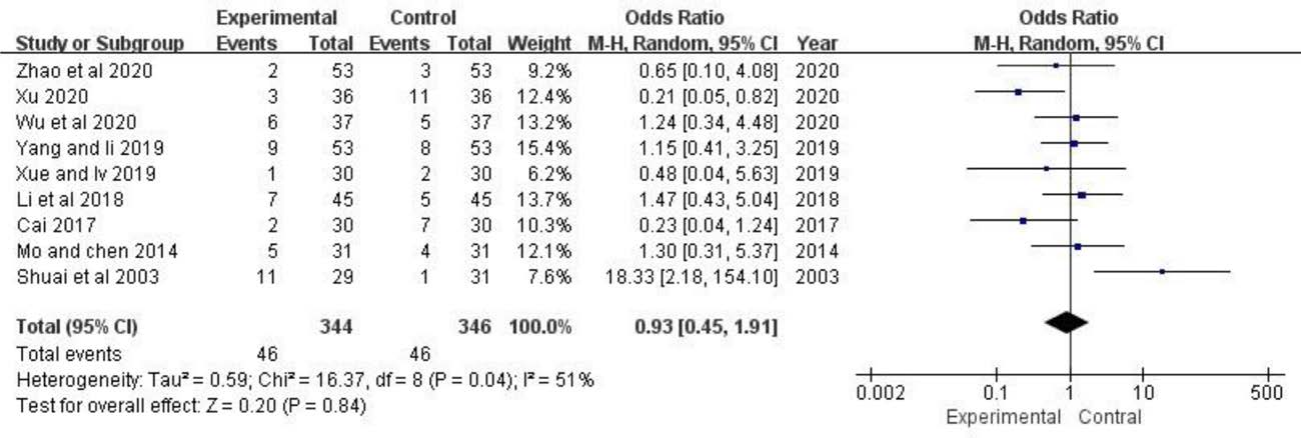
The effect of TGP on the incidence of adverse reactions. TGP = total glucosides of paeony.

A total of 92 of 690 evaluable patients from only 9 studies^[21–27,29,31]^ experienced adverse reactions. The random-effect model was adapted owing to the heterogeneity test (*P *= .04, *I*^2* *^= 51%). The results showed that the incidence of adverse reactions in the TGP group and the control group were equal with no statistical difference (OR* *= 0.93, 95% CI: 0.45 to 1.91, *Z *= 0.20, *P *= .84) (Fig. 13). It is suggested that the combined use of TGP did not increase the incidence of adverse reactions.

Our review revealed a more comprehensive evaluation of safety than the previous analysis. Two trials^[28,30]^ did not mention adverse reactions, and 9 trials^[21–27,29,31]^ mentioned adverse reactions, including 46 cases in TGP group and 46 cases in control group (Table 2). In all studies, no significant life-threatening events occurred.

**Table 2:** The effect of TGP on adverse reactions.

Included literature	Adverse reaction	
	Experimental group	Control group
Wu et al 2020	Menstrual disorders in 3 cases, intestinal reaction in 2 cases, hypertension in 1 case	Menstrual disorders in 2 cases, intestinal reaction in 2 cases, hypertension 1 case
Xu 2020	Urinary tract infection in 2 cases, pulmonary infection in 1 case	Urinary tract infection in 5 cases, pulmonary infection in 2 cases, fungal infection in 4 cases
Zhao et al 2020	Diarrhea in 1 case, fever in 1 case	Diarrhea in 2 cases, fever in 1 case
Xue and lv 2019	Diarrhea in 1 case	Diarrhea in 1 case, fever in 1 case
Yang and li 2019	Diarrhea in 4 cases, osteoporosis in 1 case, loss of appetite in 2 cases, acne in 2 cases	Osteoporosis in 2 cases, loss of appetite in 2 cases, acne in 3 cases, adrenocortical hyperfunction in 1 case
Li et al 2018	Diarrhea in 2 cases, nausea in two cases, fever in 3 cases	Diarrhea in 1 case, nausea in 2 cases, fever in 2 cases
Cai 2017	Infection in 1 case, abdominal discomfort in 1 case	Infection in 1 case, abdominal discomfort in 1 case, dizziness in 1 case, nausea in 1 case
Mo and chen 2014	Diarrhea in 2 cases, abdominal distension, nausea, and anorexia in 3 cases	Abdominal distension, nausea, and anorexia in 4 cases
Shuai et al 2003	Diarrhea in 6 cases, abdominal pain in 3 cases, anorexia, and nausea in 2 cases	Diarrhea in 1 case

TGP = total glucosides of paeony.

## 4. Discussion

Our meta-analysis indicates that TGP had an important role in the treatment of SLE in numerous ways. The projected incidence of 23.2 cases per 100,000 individuals in North America is the highest in the globe.^[32]^ African Americans, Hispanics, and Asians are more likely to have it than whites.^[33]^ Moreover, mortality is still 2 to 3 times greater in patients with SLE than in the general population, especially among females, members of racial/ethnic minority groups, dwellers of the South or West, and persons aged 65 or older.^[34,35]^ In modern medicine, glucocorticoids and immunosuppressants are commonly utilized, but they have severe adverse effects. TCM has played an increasingly significant role in the treatment of SLE in recent years. TCM has distinctive advantages and qualities for treating patients, decreasing adverse effects to chemical medication, and controlling the immune system.^[36–38]^

There is no record of any description of SLE in Chinese medicine. According to its clinical manifestations, it can be attributed to “Yin-Yang poison,” “warm poison hair spot,” “butterfly sore flow turbidity,” “arthralgia,” “edema,” “consumptive disease, and so on. The basic pathogenesis is characterized by “deficiency of healthy qi and pathogenic qi excess, viscera deficiency and deficiency of qi-blood.”^[39]^

Baishao first appeared in Shijing (the Book of Songs) under the name peony, and its nature, flavor, and efficacy were recorded for the first time in Shennong Bencao Jing (Shennong’s Classic of Materia Medica), which ranked first. Baishao is defined as sour, bitter, and slightly cold in the Chinese Pharmacopoeia. It enters the liver and spleen meridians and has the effects of nourishing blood, restraining yin, softening liver, easing pain, and suppressing liver yang. TGP are the total glucosides extracted from paeony decoction. TGP can control the immune system in both directions and has analgesic, liver protective, and anti-inflammatory properties. Due to the reduced adverse effects and improved tolerability of TGP in the treatment of SLE, TGP may be a suitable option for patients with early-stage autoimmunity or those who are ineligible for immunosuppressive or hormonal therapy.^[40]^

Modern studies have found that lymphopenia reduction in SLE patients is independently associated with increased neurological and organ damage.^[41]^ Animal experiments and clinical trials have confirmed that the lack of CD4 + CD25 + lymphocytes can lead to the occurrence and progression of SLE,^[42,43]^ and TGP can improve the level of CD4 + CD25 + lymphocytes and play a role in treating and reducing disease recurrence.^[44]^ Interferon-alpha (INF-α) played a fundamental role in the pathogenesis of SLE,^[45]^ and TGP can inhibit the secretion of interferon-alpha from peripheral blood mononuclear cells, thereby controlling the progression of SLE.^[46]^ In addition, some researches indicated that estrogen receptor alpha (ERα) signaling in B cells specifically promotes SLE. Autoantibody generation and immune cell activation were drastically reduced when ERα was deleted from B cells.^[47]^ In mouse models of SLE, ERα insufficiency significantly reduced disease severity and increased survival.^[48]^ Liu’s findings suggested that estrogen and demethylated ERα promoter associated up-regulated ERα genes are 2 critical factors in the gender-biased development of autoimmune diseases in addition to genetic factor.^[49]^ Lower genomic methylation levels in female SLE patients result in ER overexpression. One study showed that TGP increased ERα promoter DNA methylation levels and decreased ERα mRNA and protein levels in SLE mice spleen tissues.^[50]^ Moreover, IL-6 levels in serum and urine are significantly enhanced and correlate with disease activity in SLE.^[51]^ TGP can decrease IL-6 levels and the inflammatory response.^[48]^

This study aims to thoroughly assess the efficacy and safety of TGP in treating SLE. The results of the meta-analysis demonstrated that TGP in conjunction with conventional treatment can boost clinical efficacy, decrease SLEDAI score, decrease ESR and serum immunoglobulin (IgA, IgG, and IgM) levels, and enhance complement levels (C3 and C4).The reduction of recurrence rate was better than that of the control group. However, the results were highly heterogeneous in reducing the SLEDAI score. Analyses of sensitivity revealed that there may be differences in the efficacy of TGP when combined with various medicines for the treatment of SLE, which should be approached with caution. The incidence of adverse effects did not differ significantly between the 2 groups. The results demonstrate that TGP can improve treatment efficacy, reduce the recurrence rate, reduce the inflammatory response, and reduce or prevent organ and system damage, providing a theoretical basis for the treatment of SLE with an interdisciplinary approach combining traditional Chinese and western medicine.

However, the shortcomings of TGP cannot be overlooked TGP has a moderate therapeutic impact and displays sluggish and progressive efficacy in the treatment of SLE. Therefore, TGP cannot alleviate symptoms as rapidly as synthetic medications. A combination of TGP with synthetic medications can exert a synergistic therapeutic effect, which may allow for the reduction of synthetic drug doses and the mitigation of adverse effects. In the future, randomized double-blind clinical trials between TGP alone and chemotherapy for autoimmune diseases could be evaluated.

### 4.1. Study limitations

1. The quality of RCTs included in this study is still not high enough. Only one article^[31]^ adopts randomized double-blind controlled trial, and the other 10 literatures^[21–30]^ do not mention double-blind. Among them, the random method of 2 literatures^[29,30]^ is not clear, and there might be publication bias.
2. The language is limited to Chinese and English, and there is a certain degree of bias in selection.
3. In the available literature, diagnostic criteria were defined by 3 reputable institutions. In each body of literature, the standard medication, measurement, and course of treatment are different, which may result in substantial variation.
4. A small number of RCTs and patients were included, and the results were biased.

### 4.2. Expectations

This study demonstrates that the combination of TGP and Western medicine is superior to western medicine alone. Our study has a modest sample size, and the majority are exploratory clinical investigations. Due to the dearth of large-sample, multicenter, randomized, double-blind, placebo-controlled parallel trials, the general quality of the scientific literature is inadequate. We anticipate further high-quality clinical investigations. In addition, the majority of RCTs of TGP lasted between 3 and 6 months, lacked long-term follow-up, and did not assess the long-term efficacy of TCM. A number of studies failed to disclose the trials in a standardized manner, and it is recommended that, in the future, clinical trials be reported in strict accordance with the CONSORT reporting guidelines.

## Acknowledgments

All authors have completed the ICMJE uniform disclosure form at http://www.icmje.org/coi_disclosure.pdf and declare: no support from any organization for the submitted work, no financial relationships with any organization that might have an interest in the submitted work in the previous 3 years, no other relationships or activities that could appear to have influenced the submitted work.

## Author contributions

**Conceptualization:** Mengjie Wang, Zhiyuan Wang, Ying Liu, Ping Jiang.

**Data curation:** Mengjie Wang, Zhiyuan Wang, Lei Wang, Xiaomeng Wang.

**Formal analysis:** Mengjie Wang, Zhiyuan Wang.

**Funding acquisition:** Ying Liu, Ping Jiang.

**Writing-orginal draft:** Mengjie Wang, Zhiyuan Wang.

**Writing-review&editing:** Mengjie Wang, Zhiyuan Wang, Ying Liu.
